# International comparative study of learning trajectories based on TIMSS 2019 G4 data on cognitive diagnostic models

**DOI:** 10.3389/fpsyg.2023.1241656

**Published:** 2023-10-27

**Authors:** Zhemin Zhu

**Affiliations:** Beihua University, Jilin, China

**Keywords:** TIMSS, cognitive diagnosis, learning trajectories, international comparison, mathematics education

## Abstract

Learning trajectory describes the student’s learning progress or steps in one area through which teaching and learning can be linked. The cognitive diagnostic model (CDM) is an emerging evaluation theory in education measures. Researchers can determine students’ mastery of fine-grained knowledge points by describing the learning trajectory based on CDM. The present study is based on the Trends in International Mathematics and Science Study (TIMSS) 2019, particularly, 21 mathematical items in the fourth grade. It analyzes the response data of 2,854 students from 17 countries from 10 attributes based on CDM. This study explores students’ different learning trajectories in the content field by analyzing the relationships between knowledge states and attribute patterns. The study found that the 17 countries differ in learning trajectories but have commonalities. The learning starting points of 17 countries can be roughly divided into two categories and geometry attribute and fractions and decimals attribute are the last two attributes to master.

## Introduction

The International Association for the Evaluation of Educational Achievement (IEA) is one of the leading associations focused on evaluating student achievement levels worldwide. The Trends in International Mathematics and Science Study (TIMSS), first administered by the IEA in 1995, provides reliable and valuable data on the mathematics and science achievements of students in participating countries. TIMSS has been administered every 4 years since then. The study evaluates students’ knowledge and skills in mathematics and science and gathers information about their home and school environments. TIMSS employs various methods to assess student achievement, including questionnaires for students, teachers, and schools. The study also collects data on students’ home and school environments. TIMSS provides participating countries with comparative data to help them re-evaluate their students’ learning. TIMSS informs educational policy and highlights similarities and differences among countries, allowing participating countries to share experiences regarding the quantity and quality of student learning (Shannag et al., 2013). Evaluating mathematical achievement is an important component of TIMSS. Mathematical knowledge and competence are essential because they are fundamental to the development of science and technology (Kusmaryono, 2014) and relate to people’s everyday activities and transactions (Rudhumbu and Rudhumbu, 2018). Mathematics can also help promote critical thinking skills, reasoning, and problem-solving (Firdaus et al., 2015). Mathematics, as one of the core tests of TIMSS, has also been widely studied. For example, research data collected using TIMSS reveal that girls in Kuwait outperformed boys in Mathematics (Al-Mutawa et al., 2021), students’ achievement in TIMSS has a significant linear relationship with variables related to school factors (Wardat et al., 2022), and the relationship between socioeconomic status (SES) and TIMSS-results in mathematics has two mediators among Norwegian fifth graders (Østbø and Zachrisson, 2022). These studies either focus on the factors that affect TIMSS scores or the impact of TIMSS scores on society and education, but few studies analyze TIMSS projects, especially exploring the deep-level mining of information presented by Mathematics Content Domains in TIMSS. Relevant research has been conducted under the Program for International Student Assessment (PISA) to explore students’ learning trajectories (see Wu et al., 2020; Jia et al., 2021).

A learning trajectory describes the sequence of knowledge points in a particular field. It is a powerful tool for guiding teachers’ instruction and helping students learn. From a learning perspective, a learning trajectory reflects natural developmental progressions identified in theoretically and empirically grounded models of students’ thinking, learning, and development (Carpenter and Moser, 1984; Griffin and Case, 1997). Thus, learning based on a learning trajectory is efficient. From a teaching perspective, a learning trajectory provides a scientific teaching sequence; learning consistent with this trajectory is believed to be more effective for students than learning that does not follow these trajectories (Clements and Sarama, 2004). Therefore, exploring the learning sequence of mathematical knowledge is one way to help students learn effectively.

This study follows the existing CDM to seek the learning trajectory of mathematics knowledge. The study uses it as an analytic tool to analyze the TIMSS data set consisting of 17 countries and regions, namely, Armenia, Australia, Azerbaijan, Bahrain, Belgium, Bulgaria, Cyprus, *Islamic Rep. of Iran*, Ireland, Japan, Kazakhstan, Latvia, New Zealand, Northern Ireland, Oman, Poland, and Serbia on the mathematics items in the TIMSS test contents. First, the research about learning trajectory and cognitive diagnosis models are introduced. Second, the process of analyzing data is introduced. Third, the study provides the trajectory of statistical content learning in different countries. Finally, the learning trajectory is analyzed.

## Research about learning trajectory

Learning trajectory can be obtained through continuous observation of individual learning processes or through quantitative research on many students’ tests (Jia et al., 2021). The research in this field first started in the field of science and then gradually expanded to various disciplines. However, in some mathematics education studies, learning progressions and learning trajectory have no clear distinction, meaning learning progressions are virtually synonymous with learning trajectory (Empson, 2011). The following text uses learning trajectory to express.

The research on learning trajectory has a long history and has achieved a series of results. English (1991) studied discrete task combinations in Australia to find a learning trajectory for probabilistic learning in 1988. The results were first published in 1991. In this study, six solution strategies were revealed, which were used by children in solving permutation and combination problems based on an analysis of their performance, ranging from a random selection of items to systematic patterns of item selection. In 1993, the spontaneous application of strategies by children aged 7–12 was investigated when solving new combination problems. Specifically, the turning point in the changing perception of probability was found in his research’s progression of children’s strategies (English, 1993). The basic approach of this study was to assign a set of six problems to each child individually, including combinations of various possible shirts and pants (two-dimensional) or shirts, pants, and tennis rackets (three-dimensional) for teddy bears. The turning point of children’s cognition was sought by studying the number of problem solvers under different ages and strategies. This study continued until 2007 (English, 2007). Following this, another study compared the mathematics performance of eighth-grade students in 20 countries using data from TIMSS-R-1999. The study found that students in Singapore, South Korea, and Hong Kong achieved high grade point averages primarily because of their proficiency in algebra skills and complex problem-solving abilities (Tatsuoka et al., 2004). This study followed English’s line of thought but used a rule space model (RSM) for analysis. In 2008, another research compared groups of students participating in TIMSS (Dogan and Tatsuoka, 2008). This study was the first to identify cognitive skills (attributes). The 162 items of TIMSS were coded according to their attribute participation, and a Q-matrix was created. The Q-matrix and student response data determined each student’s attribute mastery profile. Turkish students’ average attribute mastery level was then calculated and compared with their American peers. The methods and procedures of this study were nearly identical to those used by Tatsuoka in 2004. In 2020, another study inherited Dogan’s ideas and used PISA test data for learning trajectory. This study used CDM instead of RSM. The result showed that students from Australia, Canada, the United Kingdom, and Russia shared similar main learning trajectories, whereas Finland and Japan were consistent with their main learning trajectories (Wu et al., 2020). Subsequently, a study used the same method with PISA data to study learning trajectories in statistics (Jia et al., 2021). These studies follow a similar line of thought but with improved methods. The basic idea of these studies is to rank the probability or accuracy of students’ mastery of knowledge points. The sequence is based on the principle of simple knowledge points before difficult knowledge points. However, the methods used in these studies are different, starting from the accuracy rate of questions based on the initial basis, then based on the Item response theory, and finally based on CDM. While recent research has utilized data from PISA, less research has been conducted using data from TIMSS.

## Cognitive diagnostic model

CDM is the latest educational and psychometric method developed from item response theory. The construction of the CDM is based on two elements. One element is an item and attribute association matrix called Q-matrix (Tatsuoka, 1983); another is a list of models used to identify students’ potential cognitive characteristics or skill mastery patterns (Jia et al., 2021).

Q-matrix designed by experts or estimated from data is a matrix that associates each item in a test with the cognitive skills required to answer it correctly. In the Q matrix, each row represents an item, and each column represents an attribute. The Q-matrix gives information on which cognitive attributes were examined for each item.


$$Q=\left(\begin{array}{ccc} 1 & 1 & 1\\ 1 & 0 & 1 \end{array}\right),$$

which indicates two items examined with three attributes. The first item examines the total three attributes, whereas the second item examines the first and third attributes. For each item, the attribute checked is represented by 1, and vice versa is represented by 0. In other words, both items were examined for the first and third attributes. The second attribute is only tested by the first item. An attribute pattern is a vector of whether a candidate has mastered the test attribute. Attribute pattern (1, 0, 1) means the examinees masters the first and the third attribute. Corresponding to the Q matrix above, this examinee is likely to get the second item right but also likely to get the first item wrong. The Q matrix gives the information of the item, while the response contains the information of the students. The two are linked through the cognitive diagnostic model.

The cognitive diagnostic model reflects the process of students’ answering and explains the relationship between students’ mastery of attributes pattern, Q matrix, and responses. Models have different types: compensatory, non-compensatory, and general models. They reflect three different cognitive processes. Different properties in the compensatory model can produce substitution effects. In other words, as long as a property is satisfied, the correct response can be generated. For the above Q-matrix, if the examinee masters only the first attribute, then the correct response will be produced on both items. Non-compensatory models are just the opposite. Examinees must master all item abilities to be able to get them right. The general model considers both cases at the same time.

The most famous compensatory model is the deterministic input, noisy-or-gate (DINO) model (Templin and Henson, 2006). The most famous non-compensatory model is the deterministic input noisy and gate (DINA) model (Junker and Sijtsma, 2001), and the most famous general model is the general DINA (G-DINA) model (de la Torre, 2011). Some other models, such as the Reduced Reparameterized Unified Model (Hartz et al., 2002), the liner logistic model (Maris, 1999), the log-linear model with latent variables for cognitive diagnosis (Henson et al., 2009), and the general diagnostic model (GDM; von Davier, 2005). G-DINA is the general form of DINA and DINO, whereas GDM is the more general form of G-DINA. GDM can degenerate into G-DINA, whereas G-DINA can degenerate into DINA and DINO. These models explain different cognitive processes. However, researchers often cannot know the real cognitive process of examinees, so they rely more on model selection to choose the most appropriate cognitive diagnostic model. According to the Q matrix, cognitive diagnostic model, and responses, the student’s answering process is explained, and the student’s attribute pattern can be inferred by statistical methods.

## Materials and methods

### Participants

Data from the TIMSS 2019 Mathematics Exam was selected for this study. All data can be downloaded from the TIMSS public database. This study is based on the data of 2,854 students in fourth grade from 17 countries and regions, namely, Armenia, Australia, Azerbaijan, Bahrain, Belgium, Bulgaria, Cyprus, Islamic Rep. of Iran, Ireland, Japan, Kazakhstan, Latvia, New Zealand, Northern Ireland, Oman, Poland, and Serbia. Given that students in each country partially responded, students with missing data were removed from the analysis.

### Q matrix

A total of 21 topics were selected for this study. These items are MP71013, MP71026, MP71036A, MP71036B, MP71036, MP71075A, MP71075B, MP71080, MP71178C, MP71178, MP71135B, MP71175A, MP71175B, MP71175C, MP51206, MP51049, MP51045, MP51098, MP51030, MP51533, and MP51080. All topics can be found on TIMSS official website. These items were selected because the items were examined by all the 17 countries. On the other hand, the data analysis results show a good fitting.

Attributes are the most important roles in cognitive diagnostic measures. The quality of attributes is directly related to the effectiveness of cognitive diagnostic evaluation. To some extent, the essence of cognitive diagnosis is the measurement of cognitive attributes. Attributes can be obtained through expert discussions or data analysis. TIMSS is an international public test, and its attribute division has been completed and verified by a series of studies. According to the definitions of cognitive attributes and the test items provided by the TIMSS 2019 assessment framework, each item in this study is defined from two dimensions: topic area and cognitive domain (IEA, 2019). The attributes in each dimension and the corresponding definitions are shown in Table 1.

**Table 1:** Dimensions of cognitive attributes.

Dimension	Attribute	No.	Definition
Topic area	Whole Numbers	T1	Demonstrate knowledge of place value; represent whole numbers with words, diagrams, number lines, or symbols; order numbers, add and subtract, multiply, and divide, solve problems involving odd and even numbers, multiples, and factors of numbers, rounding numbers, making estimates, and combining two or more properties of numbers or operations to solve problems in context
	Expressions, Simple Equations, and Relationships	T2	Find the missing number or operation in a number sentence, identify or write expressions or number sentences to represent problem situations that may involve unknowns, identify and use relationships in a well-defined pattern
	Fractions and Decimals	T3	Recognize fractions as parts of wholes or collections; represent fractions using words, numbers, or models; compare and order simple fractions; add and subtract simple fractions, including those set in problem situations, demonstrate knowledge of decimal place value including representing decimals using words, numbers, or models; compare, order, and round decimals; add and subtract decimals, including those set in problem situations
	Measurement	T4	Measure and estimate lengths, solve problems involving lengths, solve problems involving mass, volume, and time, identify appropriate types and sizes of units and read scales, solve problems involving perimeters of polygons, areas of rectangles, areas of shapes covered with squares or partial squares, and volumes filled with cubes
	Geometry	T5	Identify and draw parallel and perpendicular lines; identify and draw right angles and angles smaller or larger than a right angle; compare angles by size, use elementary properties, including line and rotational symmetry, to describe, compare, and create common two-dimensional shapes, use elementary properties to describe and compare three-dimensional shapes and relate these with their two-dimensional representations
	Reading, Interpreting, and Representing	T6	Read and interpret data from tables, pictographs, bar graphs, line graphs, and pie charts, organize and represent data to help answer questions
	Using Data to Solve Problems	T7	Use data to answer questions that go beyond directly reading data displays
Cognitive domain	Knowing	C1	Recall; Recognize; Classify/Order; Compute; Retrieve; Measure
	Applying	C2	Determine; Represent/Model; Implement
	Reasoning	C3	Analyze; Integrate/Synthesize; Evaluate; Draw Conclusions; Generalize; Justify

In Table 1, the topic area includes all the content learned in the fourth grade of primary school, whereas the cognitive domain divides the cognitive difficulty. These two dimensions include students’ learning content and cognitive level. Based on the questions and cognitive attributes, a Q matrix can be developed for these 21 questions and 10 attributes. The Q-matrix is obtained, as shown in Table 2.

**Table 2:** Q-matrix of 21 test items in TIMSS.

	Topic Area							Cognitive Domain		
	T1	T2	T3	T4	T5	T6	T7	C1	C2	C3
MP71013	1	0	0	0	0	0	0	1	0	0
MP71026	1	0	0	0	0	0	0	0	0	1
MP71036A	0	1	0	0	0	0	0	0	1	0
MP71036B	0	1	0	0	0	0	0	0	1	0
MP71036	0	1	0	0	0	0	0	0	1	0
MP71075A	0	0	0	1	0	0	0	0	1	0
MP71075B	0	0	0	1	0	0	0	0	1	0
MP71080	0	0	0	1	0	0	0	0	0	1
MP71178C	0	0	0	0	1	0	0	1	0	0
MP71178	0	0	0	0	1	0	0	1	0	0
MP71135B	0	0	0	0	0	1	0	1	0	0
MP71175A	0	0	0	0	0	0	1	0	1	0
MP71175B	0	0	0	0	0	0	1	0	1	0
MP71175C	0	0	0	0	0	0	1	0	1	0
MP51206	1	0	0	0	0	0	0	1	0	0
MP51049	1	0	0	0	0	0	0	0	1	0
MP51045	0	0	0	1	0	0	0	0	1	0
MP51098	0	0	1	0	0	0	0	1	0	0
MP51030	0	0	1	0	0	0	0	0	1	0
MP51533	0	0	0	1	0	0	0	0	0	1
MP51080	0	0	0	0	0	1	0	0	1	0

The Q matrix in Table 2 shows which attributes are tested for each item and which items tested the attributes. These attributes are marked by TIMSS, but further analysis is needed to determine whether the data is fitted. Whether the data and model fit or not requires a two-step test, relative fit and absolute fit indicators.

### Model selection

Model selection (relative fit) is one of the important steps in using cognitive diagnosis. The result of model selection determines which model fits the data better. Essentially, model selection involves finding suitable explanations for students’ cognitive processes through data. After model selection, the data quality can be further verified based on the selected model.

Many cognitive diagnosis practices have shown that choosing an appropriate cognitive diagnostic model is an important prerequisite for accurately diagnosing or classifying subjects (Tatsuoka, 1984). The most popular reference standards are Akaike’s information criterion (AIC) and Bayesian information criterion (BIC). Both are used in this study. The model with the smallest AIC and BIC should be chosen. The result is shown in Table 3.

**Table 3:** The result of model selection.

	Deviation	AIC	BIC
DINA	60639.90	62769.90	69113.55
DINO	60649.28	62779.28	69122.93
rRUM	59951.79	62123.79	68592.52
LLM	59731.60	61903.60	68372.34
ACDM	59726.84	61898.84	68367.58
GDM	60003.66	60241.66	60950.48
LCDM	60010.62	60290.62	61124.53
GDINA	59514.05	61728.05	68321.86

Table 3 shows that GDM has the minimum in AIC and BIC. Thus, GDM should be chosen as the model.

### Instrument

After determining the model, the quality of the test questions must be further evaluated. This process involves the following steps.

Model selection can only indicate that the GDM model fits the data better than other models. However, the fitting effect needs to be tested using item fitting (absolute fit indicators). The Root Mean Square Error of Approximation (RMSEA) is often used as an evaluation standard. The closer the value of RMSEA is to 0, the better the fitting of the project is. If it is less than 0.1, it is generally considered that the effect is very good (Oliveri and von Davier, 2011). The RMSEA of 21 items are shown in Table 4.

**Table 4:** The RMSEA of 21 items.

Item	RMSEA	Item	RMSEA	Item	RMSEA
Item1	0.0561	Item8	0.0667	Item15	0.0637
Item2	0.0560	Item9	0.0564	Item16	0.0536
Item3	0.0587	Item10	0.0000	Item17	0.0632
Item4	0.0730	Item11	0.0566	Item18	0.0585
Item5	0.0000	Item12	0.0429	Item19	0.0580
Item6	0.0403	Item13	0.0343	Item20	0.0528
Item7	0.0337	Item14	0.0656	Item21	0.0587

Table 4 shows that RMSEA of all 21 items is less than 0.1, which means the item fits the model very well.

Reliability represents the credibility of the exam. Two commonly used reliability are Cronbach’s (α) coefficient under classic evaluation theory (CTT) and the consistency of the retest of attributes (Templin and Bradshaw, 2013). These data indicators can be obtained through the flexCDMs analysis platform (Tu, 2019). The result shows reliability Cronbach’s (α) is 0.8707, indicating high reliability under CTT theory. The reliability index of 10 attributes by Templin and Bradshaw, 2013 are 0.99, 0.8523, 0.99, 0.7035, 0.8599, 0.99, 0.7919, 0.9178, 0.8574, 0.8887, 0.8813, and 0.8753. If the reliability indexes of attribute is greater than 0.7, it generally has high reliability (Wu et al., 2020). So, the attributes have high reliability.

Discrimination represents whether the question can distinguish students of different levels. The discrimination *d_j_* in CDM is defined as follows:


$$d_{j}=P_{j}\;\left(1\right)-P_{j}\left(0\right),$$

where *P_j_*(1) represents the likelihood of correctly answering item *j*th when the examinee master all the attributes. Conversely, *P_j_*(0) denotes the probability of answering item *j*th correctly with none of the attributes. If the discrimination indexes of attribute is greater than 0.4, it generally has good discrimination effect (Tu, 2019). The discrimination of 21 items is as shown in Table 5.

**Table 5:** The discrimination of 21 items.

Item	Discrimination	Item	Discrimination	Item	Discrimination
Item1	0.5799	Item8	0.5640	Item15	0.5513
Item2	0.7997	Item9	0.7734	Item16	0.7068
Item3	0.7063	Item10	0.9999	Item17	0.7117
Item4	0.8113	Item11	0.5912	Item18	0.6772
Item5	0.9999	Item12	0.7621	Item19	0.5420
Item6	0.8525	Item13	0.7244	Item20	0.7177
Item7	0.7584	Item14	0.7795	Item21	0.7813

Table 5 shows that discrimination of all the 21 items are bigger than 0.4, which is only 5 discrimination less than 0.7, which means the items have a high discrimination effect.

Finally, the validity of the Q-matrix needs to be assessed. A linear regression analysis could be conducted to see if the columns of the Q-matrix can explain item difficulty (Dogan and Tatsuoka, 2008). The difficulty of 21 items by IRT is shown in Table 6.

**Table 6:** The difficulty of 21 items.

Item	Difficulty	Item	Difficulty	Item	Difficulty
Item1	−1.1390	Item8	−0.4146	Item15	−0.6939
Item2	−0.2112	Item9	−0.4702	Item16	−0.5729
Item3	−1.4085	Item10	−0.0470	Item17	−0.4658
Item4	−1.2572	Item11	−0.8852	Item18	0.0187
Item5	−0.9697	Item12	−0.5072	Item19	0.9138
Item6	−0.1310	Item13	−1.1335	Item20	−0.1183
Item7	0.4397	Item14	−0.3870	Item21	−0.4175

A linear regression analysis between the Q-matrix and difficulty was constructed. An adjusted *R*^2^ value of 0.674 was obtained, indicating that nearly 70% of the variance in item difficulty levels was due to attribute involvement. The Q matrix can better explain the difficulty of the problem and also indicate the effectiveness of the Q matrix.

## Research analysis and results

According to the above model selection results, the GDM had the best model fit. Thus, GDM was used to evaluate the parameters from the 2,854 responses. In the GDINA package of the software RStudio Version 1.4.1103, the probability of a student mastering a knowledge state can be obtained from expected *a posteriori* (EAP) through the function “personparm,” that is, the probability that a student masters a certain attribute. It is generally believed that a probability of mastery greater than 0.5 indicates that an examinee has mastered this attribute, represented by 1. If it is less than 0.5, then they have not mastered this attribute, represented by 0. Thus, the examinee’s mastery probability on each attribute and attribute pattern can be obtained.

### Comparative analysis of attribute pattern

#### Attribute pattern

The attribute pattern is a vector representing whether the student has mastered the corresponding attribute. It represents the mastery of a field of knowledge and skills, where 1 indicates that the subject has mastered the corresponding attributes, and 0 indicates that the subject has not mastered the corresponding attributes (Tatsuoka, 2009). For example, attribute pattern (1,1,0) represents that the student has mastered the first and second attributes but not the third attribute. The top 3 attribute patterns of proportion in the topic area in each country are shown in Table 7.

**Table 7:** The three most frequency patterns in Topic Area.

	Pattern	Rate	Pattern	Rate	Pattern	Rate
Armenia	(1,1,1,1,0,1,1)	0.19	(1,1,1,1,1,1,1)	0.12	(1,1,0,1,0,1,0)	0.07
Australia	(1,1,1,1,1,1,1)	0.11	(0,1,0,0,0,1,1)	0.06	(0,0,0,0,0,0,0)	0.04
Azerbaijan	(1,1,1,1,1,1,1)	0.11	(1,1,0,1,1,1,1)	0.07	(1,1,1,1,0,1,1)	0.07
Bahrain	(0,0,0,0,0,0,0)	0.09	(1,1,0,1,1,1,1)	0.05	(1,1,0,0,0,1,1)	0.04
Belgium	(1,1,1,1,1,1,1)	0.13	(1,1,1,1,1,1,0)	0.05	(1,1,1,0,1,1,1)	0.03
Bulgaria	(1,1,1,1,1,1,1)	0.18	(1,1,0,1,0,1,1)	0.08	(1,1,0,1,1,1,1)	0.07
Cyprus	(1,1,1,1,1,1,1)	0.15	(1,1,1,0,1,1,0)	0.05	(1,1,0,1,1,1,1)	0.05
Iran, Islamic Rep. of	(0,0,0,0,0,0,0)	0.11	(1,1,1,1,1,1,1)	0.04	(0,1,1,1,0,1,0)	0.04
Ireland	(1,1,1,1,1,1,1)	0.15	(1,1,1,0,1,1,1)	0.07	(1,1,1,1,0,1,1)	0.07
Japan	(1,1,1,1,1,1,1)	0.20	(0,1,0,1,0,1,1)	0.06	(0,1,1,1,1,1,1)	0.06
Kazakhstan	(1,1,1,1,0,0,1)	0.05	(0,1,1,1,1,1,1)	0.04	(0,1,1,1,0,1,1)	0.04
Latvia	(1,1,1,1,1,1,1)	0.13	(1,1,0,1,1,1,1)	0.12	(0,1,1,1,1,1,1)	0.05
New Zealand	(0,0,0,0,0,0,0)	0.09	(1,1,1,1,1,1,1)	0.06	(0,0,0,0,0,0,1)	0.04
Northern Ireland	(1,1,1,1,1,1,1)	0.27	(1,1,0,1,1,1,1)	0.05	(1,1,1,1,0,1,1)	0.05
Oman	(0,0,0,0,0,0,0)	0.16	(0,0,1,0,0,0,0)	0.05	(0,1,0,0,0,0,0)	0.05
Poland	(1,1,1,1,1,1,1)	0.15	(0,1,1,1,1,1,1)	0.07	(1,1,0,1,1,1,1)	0.06
Serbia	(1,1,1,1,1,1,1)	0.12	(1,1,0,1,1,1,1)	0.11	(1,1,1,1,1,0,1)	0.07

Table 7 indicates that 12 countries, Australia, Azerbaijan, Belgium, Bulgaria, Cyprus, Ireland, Japan, Latvia, Northern Ireland, Poland, and Serbia, ranked first (1,1,1,1,1,1,1,). It shows these countries have a better attribute grasp of the topic area. Bahrain, Iran, Islamic Rep. Of, New Zealand, and Oman ranked first (0,0,0,0,0,0,0), which may mean that mathematics learning levels of students in these countries need to be improved. Geometry attribute and fractions and decimals attribute may be the least two attributes to be mastered. The top 3 attribute patterns of proportion in the cognitive domain in each country are shown in Table 8.

**Table 8:** The three most frequency patterns in Cognitive Domain.

	Pattern	Rate	Pattern	Rate	Pattern	Rate
Armenia	(1,1,1)	0.49	(0,1,1)	0.16	(1,1,0)	0.14
Australia	(0,0,0)	0.26	(1,1,1)	0.17	(0,0,1)	0.15
Azerbaijan	(1,1,1)	0.37	(0,0,0)	0.14	(0,1,1)	0.14
Bahrain	(0,0,0)	0.28	(1,0,0)	0.18	(0,0,1)	0.16
Belgium	(1,1,1)	0.26	(0,0,0)	0.18	(0,1,0)	0.17
Bulgaria	(1,1,1)	0.41	(1,1,0)	0.16	(1,0,0)	0.11
Cyprus	(0,1,1)	0.28	(0,0,0)	0.15	(0,0,1)	0.14
Iran, Islamic Rep. of	(1,1,0)	0.22	(0,0,0)	0.20	(0,1,1)	0.12
Ireland	(1,1,1)	0.33	(1,1,0)	0.16	(0,1,0)	0.11
Japan	(1,1,1)	0.59	(1,1,0)	0.11	(0,1,1)	0.09
Kazakhstan	(1,1,1)	0.31	(1,0,0)	0.15	(1,1,0)	0.14
Latvia	(1,1,1)	0.43	(1,1,0)	0.13	(0,0,0)	0.12
New Zealand	(0,0,0)	0.25	(1,1,1)	0.20	(0,0,1)	0.12
Northern Ireland	(1,1,1)	0.30	(1,1,0)	0.22	(0,1,0)	0.11
Oman	(0,0,0)	0.33	(0,0,1)	0.24	(1,0,1)	0.11
Poland	(1,1,1)	0.30	(0,1,1)	0.14	(1,0,1)	0.13
Serbia	(1,1,1)	0.39	(1,1,0)	0.18	(1,0,0)	0.17

Table 8 indicates that 17 countries are quite differentiated in the cognitive domain. Nearly half of the students in Armenia mastered all three attributes, but almost one-third of all the students in Australia, Bahrain, New Zealand, and Oman have not mastered any attribute in the cognitive domain dimension. Bulgaria and Serbia show the same learning trajectory. The three most frequent patterns in cognitive domain are (1,1,1), (1,1,0), and (1,0,0). The three most frequency patterns of Ireland are (0,1,0), (1,1,0), and (1,1,1), reflecting differences across countries. This finding is also consistent with the existing research results (see Wu et al., 2020; Jia et al., 2021).

### Comparative of attribute mastery probability

The probability of attribute mastery represents the degree to which students master a certain attribute. The closer the probability of mastery is to 1, the better the mastery; vice versa, the worse. Based on the estimated mastery probability of each student on each attribute in the previous step, the mastery probability of each country on each attribute can be calculated, which is expressed as an average. The specific results are shown in Table 9.

**Table 9:** Proportional distribution of 10 attributes in 17 countries.

	Topic Area							Cognitive Domain		
	T1	T2	T3	T4	T5	T6	T7	C1	C2	C3
Armenia	0.89	0.87	0.59	0.92	0.50	0.88	0.83	0.72	0.81	0.72
Australia	0.62	0.74	0.33	0.63	0.51	0.80	0.82	0.34	0.41	0.39
Azerbaijan	0.80	0.81	0.31	0.80	0.42	0.72	0.71	0.60	0.64	0.49
Bahrain	0.58	0.54	0.24	0.54	0.38	0.63	0.61	0.21	0.25	0.20
Belgium	0.71	0.66	0.41	0.71	0.58	0.77	0.74	0.46	0.55	0.47
Bulgaria	0.87	0.83	0.37	0.88	0.59	0.84	0.85	0.64	0.67	0.58
Cyprus	0.79	0.82	0.50	0.66	0.55	0.77	0.77	0.37	0.54	0.45
Iran, Islamic Rep. of	0.61	0.64	0.26	0.61	0.36	0.61	0.56	0.30	0.37	0.25
Ireland	0.82	0.75	0.49	0.71	0.58	0.80	0.82	0.50	0.63	0.54
Japan	0.84	0.86	0.43	0.92	0.66	0.87	0.88	0.76	0.80	0.70
Kazakhstan	0.77	0.73	0.29	0.81	0.39	0.69	0.72	0.52	0.55	0.37
Latvia	0.76	0.81	0.28	0.80	0.63	0.78	0.82	0.61	0.65	0.55
New Zealand	0.55	0.65	0.28	0.53	0.40	0.68	0.69	0.32	0.38	0.34
Northern Ireland	0.85	0.74	0.55	0.79	0.70	0.83	0.84	0.57	0.67	0.59
Oman	0.38	0.51	0.19	0.36	0.31	0.47	0.40	0.15	0.19	0.17
Poland	0.67	0.79	0.35	0.76	0.64	0.81	0.81	0.53	0.58	0.51
Serbia	0.82	0.87	0.34	0.83	0.49	0.76	0.76	0.63	0.66	0.54

**Figure 1:**
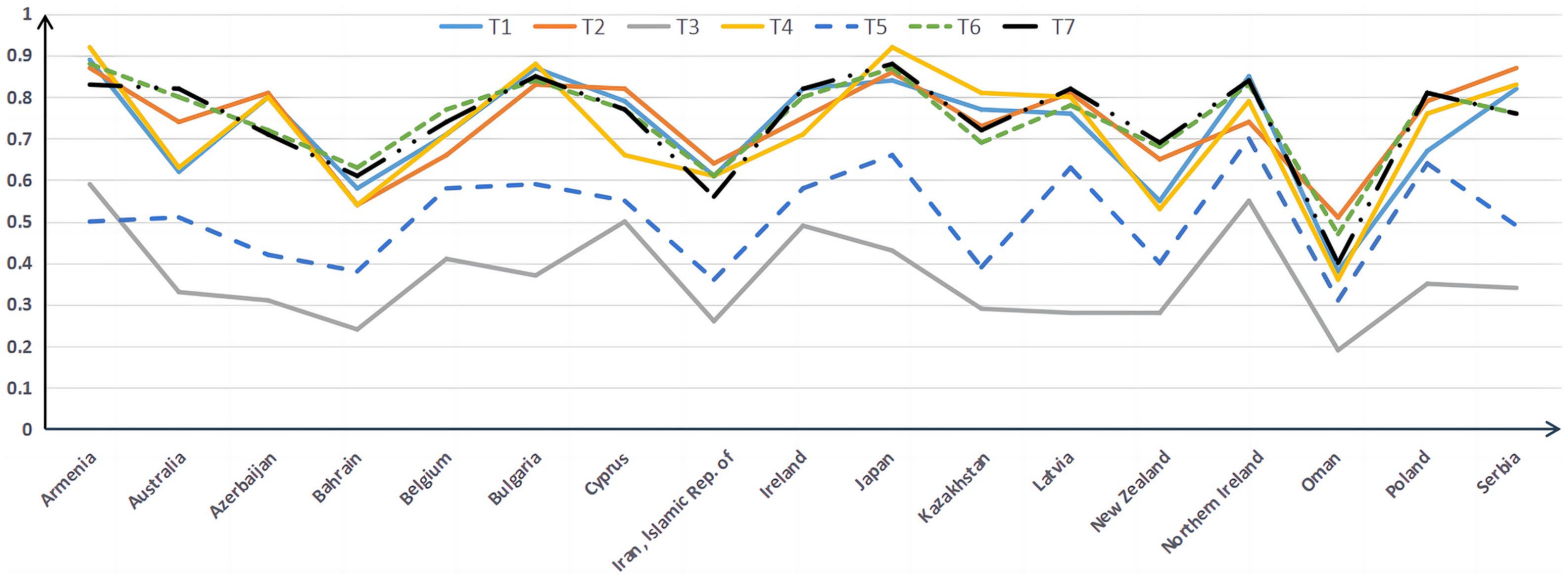
Probability distribution map of Topic Area in 17 countries.

**Figure 2:**
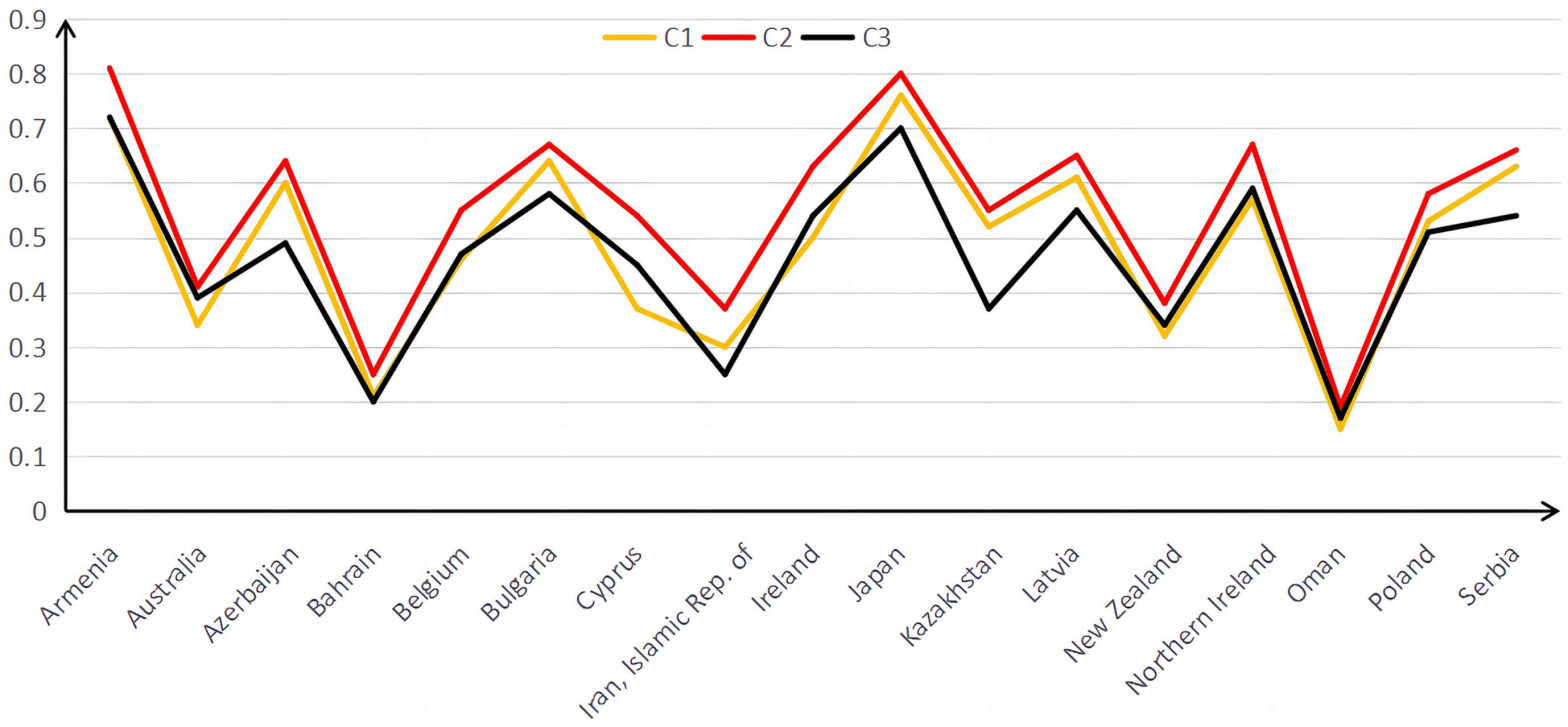
Probability distribution map of Cognitive Domain in 17 countries.

Table 9 shows differences in the average mastery probability of the same attributes among different countries. The same country has different average mastery probabilities on different attributes. The differences in the average mastery probability of different countries on Topic Area and Cognitive Domain are shown in Figures 1, 2.

Figure 1 indicates a similar trend in the average mastery probability across the seven attributes for all countries. T3 *Fractions and Decimals* is the worst attribute for students in all countries, followed by T5 *Geometry*, whereas the remaining attributes are similar. Examinees of Oman are not good in all attributes, whereas Armenia and Northern Ireland are good in seven. Armenia, Japan, and Bulgaria have the highest average mastery probability on the T4 *Measurement* attribute among the seven attributes. Australia, Ireland, and Poland have the highest average probability of mastering the T7 *Using Data to Solve Problems* attribute among the seven attributes. Globally, algebra and measurement questions were significantly more difficult than number, geometry, and data (OECD, 2010). However, Figure 1 indicates that T4 *Measurement* is not the worst attribute to master.

Figure 2 indicates that examinees of Oman is not good in all cognitive domain attributes. From experience, the order of difficulty of C1 *Knowing*, C2 *Applying*, and C3 *Reasoning* should be that C1 *Knowing* is the easiest, C2 *Applying* comes next, and C3 *Reasoning* is the hardest. However, Figure 2 shows that students master C2 *Applying* best. Some studies have found that the students’ mathematical operation level is weaker than that in mathematical reality (Wu et al., 2020), which is also consistent with the results found in this study.

### Learning trajectories in the topic area

**Figure 3:**
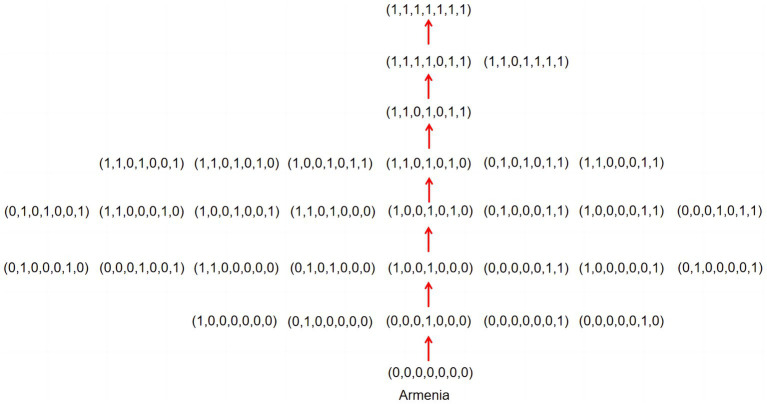
Students’ learning trajectories in Armenia.

**Figure 4:**
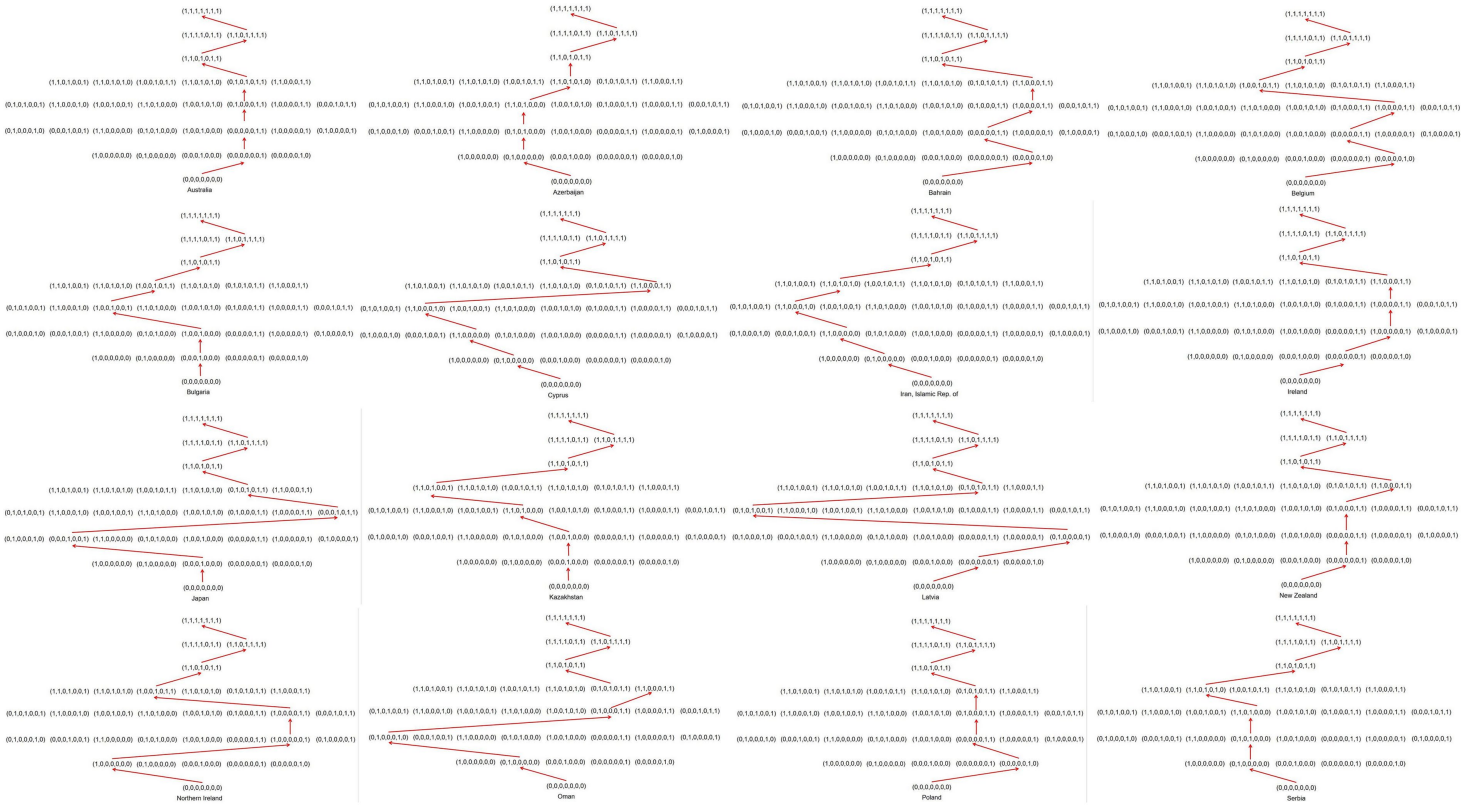
Students’ learning trajectories in 16 countries.

An important significance of learning trajectory is that it can show the order in which students learn knowledge. This order is the embodiment of students’ cognitive laws. A general belief is that students need to start learning from the simplest knowledge points (attribute) and then master advanced knowledge points (attributes). In the attribute pattern, students should gradually learn from not mastering any attributes to mastering all. The teaching or learning sequence that conforms to the cognitive rules of students’ learning can maximize learning efficiency. Confirming the progress of students’ Topic Area learning can provide a clear route for front-line teaching and precise remedial solutions for students’ learning. The biggest advantage of the cognitive diagnostic assessment is that it can grasp the cognitive laws of the subjects more deeply (Wu et al., 2020). Next, learning trajectory is constructed according to the probability of mastering each attribute in different countries for the topic area. The result is shown in Figures 3, 4.

Figures 3, 4 show the learning trajectories for the 17 countries. These trajectories are not only directly related to the cognitive order of students but also influenced by factors such as national curriculum arrangements and extracurricular tutoring (De Lange, 2007). Figure 3 shows that Armenia’s knowledge acquisition order may be T4 → T1 → T6 → T2 → T7 → T3 → T5. Although the learning trajectories vary across the 17 countries, some commonalities exist. The last two attributes Armenia masters are T3 *Fractions and Decimals* and T5 *Geometry*; students need to master T3 first and then T5. The last two attributes mastered by the other 16 countries are T5 and T3; T5 is mastered first, followed by T3. Cyprus and Iran have precisely the same route at the beginning, but the order of mastering in T6 *Reading, Interpreting, and Representing* and T7 *Using Data to Solve Problems* is reversed. Although there are differences in the starting points of learning trajectories among 17 countries, the first two attributes mastered by 5 countries are T6 *Reading, Interpreting, and Representing* and T7 *Using Data to Solve Problems*. All learning trajectories eventually converge to the same attribute pattern (1,1,0,1,0,1,1), suggesting that this pattern may be a key point in the learning process.

## Discussion

With the continuous development of globalization, more and more countries have begun to participate in nationalized examinations. TIMSS is an important exam for globalization testing in the field of mathematics. TIMSS test results have become the basis for curriculum reform in many countries. Exploring TIMSS data can discover current problems and provide new perspectives for future education. Based on the test data of TIMSS2019, this study constructed the learning trajectories of different countries and found the commonalities and differences between the learning trajectories of 17 countries. This information can provide information for teaching in different countries and comparing mathematics education between countries.

Compared with the existing research results, this study also found something. Studies have found that algebra and measurement questions were significantly more complex than numbers, geometry, and data (OECD, 2010). However, Figure 1 shows that students’ mastery of T2 *Expressions, Simple Equations, and Relationships* and T4 *Measurement* is better than T5 *Geometry*. Some research also found that uncertainty and data are the simplest content attributes (Wu et al., 2020). The research in this article supports this viewpoint. On the basis of different international tests (such as PISA and TIMSS) and different examinees, differences are observed in the analysis results. The international comparison of mathematics learning still needs to be further developed. Some studies also have shown that IQ and the economy are positively correlated (Meisenberg and Woodley, 2013). Australia has the highest GDP of 17 countries. However, judging from the probability of mastering attributes, its students are not at the forefront. As a developing country, Oman has the lowest probability of attributes mastered by its students, showing that the results of mathematics education and the country’s economic development may not necessarily be consistent.

Although this study uses CDM to conduct an in-depth analysis of the TIMSS 2019 items, some aspects still require improvement. First, the study selected 17 countries, but the total number of people was only 2,854. The reason is that not all students answer the same questions on the TIMSS exam. Therefore, candidates who have answered the same questions must be found. For CDM, having few items will lead to inaccurate estimates of mastery probability, and having too many questions makes finding candidates who can answer these questions challenging. In future research, missing data technology can be used to expand samples or increase the number of candidates that can be selected by reducing the number of attributes. Only the learning trajectory of the Topic Area is analyzed, whereas the learning trajectory of the cognitive domain is not analyzed. Most current research on mathematics learning trajectory only focuses on knowledge content, while less attention is paid to process cognitive domain. Moreover, the cognitive domain has only three attributes, and interested readers can build their own based on the data above. Finally, the effect of learning is affected by many aspects. In addition to school teaching, non-teaching factors are equally important. Future research also needs to focus on the external environment in which students learn.

## Data availability statement

Publicly available datasets were analyzed in this study. This data can be found here: TIMSS 2019.

## Author contributions

ZZ designed the study, wrote this manuscript, reviewed the manuscript, provided comments, contributed to the article, and approved the submitted version.

## References

[ref1] Al-Mutawa F. Al-Rasheedi G. Al-Maie D. (2021). Kuwaiti students’ achievements in mathematics: findings from the TIMSS assessments: reality and reasons. SAGE Open 11:215824402110319. doi: 10.1177/21582440211031903

[ref2] Carpenter T. P. Moser J. M. (1984). The acquisition of addition and subtraction concepts in grades one through three. J. Res. Math. Educ. 15, 179–202. doi: 10.2307/748348

[ref3] Clements D. H. Sarama J. (2004). Learning trajectories in mathematics education. Math. Think. Learn. 6, 81–89. doi: 10.1207/s15327833mtl0602_1, PMID: 37766757

[ref4] de la Torre J. (2011). The generalized DINA model framework. Psychometrika 76, 179–199. doi: 10.1007/s11336-011-9207-7

[ref5] De Lange J. (2007). “Large-scale assessment and mathematics education” in Second handbook of research on mathematics teaching and learning, vol. 2, 1111–1144.

[ref6] Dogan E. Tatsuoka K. (2008). An international comparison using a diagnostic testing model: Turkish students’ profile of mathematical skills on TIMSS-R. Educ. Stud. Math. 68, 263–272. doi: 10.1007/s10649-007-9099-8

[ref7] Empson S. B. (2011). On the idea of learning trajectories: promises and pitfalls. Math. Enthusiast 8, 571–596. doi: 10.54870/1551-3440.1229

[ref001] English L. D. (1991). Young children’s combinatoric strategies. Educational studies in Mathematics 22, 451–474. doi: 10.1007/BF00367908

[ref8] English L. D. (1993). Children’s strategies for solving two–and three–dimensional combinatorial problems. J. Res. Math. Educ. 24, 255–273. doi: 10.5951/jresematheduc.24.3.0255

[ref9] English L. D. (2007). “Children’s strategies for solving two-and three-dimensional combinatorial problems” in Stepping stones for the 21st century (Leiden: Brill), 139–158.

[ref10] Firdaus F. Kailani I. Bin Bakar N. Bakry B. (2015). Developing critical thinking skills of students in mathematical learning. J. Educ. Learn. 9, 226–236. doi: 10.11591/edulearn.v9i3.1830

[ref11] Griffin S. Case R. (1997). Re-thinking the primary school math curriculum: an approach based on cognitive science. Issues Educ. 3, 1–49.

[ref12] Hartz S. Roussos L. Stout W. (2002). Skills diagnosis: Theory and practice. User manual for arpeggio software. Princeton, NJ: ETS.

[ref13] Henson R. A. Templin J. L. Willse J. T. (2009). Defining a family of cognitive diagnosis models using log-linear models with latent variables. Psychometrika 74, 191–210. doi: 10.1007/s11336-008-9089-5, PMID: 23070600

[ref14] IEA (2019). TIMSS 2019 assessment frameworks IEA https://timssandpirls.bc.edu/timss2019/frameworks/framework-chapters/mathematics-framework/.

[ref15] Jia B. Zhu Z. Gao H. (2021). International comparative study of statistics learning trajectories based on PISA data on cognitive diagnostic models. Front. Psychol. 12:657858. doi: 10.3389/fpsyg.2021.657858, PMID: 34354630 PMC8329356

[ref16] Junker B. W. Sijtsma K. (2001). Cognitive assessment models with few assumptions, and connections with nonparametric item response theory. Appl. Psychol. Meas. 25, 258–272. doi: 10.1177/01466210122032064

[ref17] Kusmaryono I. (2014). The importance of mathematical power in mathematics learning. In: International Conference on Mathematics, Science, and Education (Vol. 2014, pp. 35–40). Available at: https://icmseunnes.com/2015/wp-content/uploads/2015/10/7.pdf.

[ref18] Maris E. (1999). Estimating multiple classification latent class models. Psychometrika 64, 187–212. doi: 10.1007/BF02294535, PMID: 37150103

[ref19] Meisenberg G. Woodley M. A. (2013). Are cognitive differences between countries diminishing? Evidence from TIMSS and PISA. Intelligence 41, 808–816. doi: 10.1016/j.intell.2013.03.009

[ref20] OECD (2010). Learning mathematics for life: A perspective from PISA. Paris: OECD Publishing.

[ref21] Oliveri M. E. von Davier M. (2011). Investigation of model fit and score scale comparability in international assessments. Psychol. Test Assess. Model. 53:315. Available at: https://citeseerx.ist.psu.edu/document?repid=rep1&type=pdf&doi=144bd474c2d02dfbed9e06b74f3dee015314a789.

[ref22] Østbø I. U. Zachrisson H. D. (2022). Student motivation and parental attitude as mediators for SES effects on mathematics achievement: evidence from Norway in TIMSS 2015. Scand. J. Educ. Res. 66, 808–823. doi: 10.1080/00313831.2021.1939138

[ref23] Rudhumbu N. Rudhumbu L. (2018). Implementing mathematics curriculum in primary schools in Botswana: issues and challenges. J. Stud. Soc. Sci. Hum. 4, 63–75.

[ref24] Shannag Q. A. Tairab H. Dodeen H. Abdel-Fattah F. (2013). Linking teachers’ quality and student achievement in the Kingdom of Saudi Arabia and Singapore: the impact of teachers’ background variables on student achievement. J. Balt. Sci. Educ. 12, 652–665. doi: 10.33225/jbse/13.12.652

[ref25] Tatsuoka K. K. (1983). Rule space: an approach for dealing with misconceptions based on item response theory. J. Educ. Meas. 20, 345–354. doi: 10.1111/j.1745-3984.1983.tb00212.x

[ref26] Tatsuoka K. K. (1984). Caution indices based on item response theory. Psychometrika 49, 95–110. doi: 10.1007/BF02294208, PMID: 37100805

[ref27] Tatsuoka K. K. (2009). Cognitive assessment: An introduction to the rule space method. Abingdon: Routledge.

[ref28] Tatsuoka K. K. Corter J. E. Tatsuoka C. (2004). Patterns of diagnosed mathematical content and process skills in TIMSS-R across a sample of 20 countries. Am. Educ. Res. J. 41, 901–926. doi: 10.3102/00028312041004901

[ref29] Templin J. Bradshaw L. (2013). Measuring the reliability of diagnostic classification model examinee estimates. J. Classif. 30, 251–275. doi: 10.1007/s00357-013-9129-4, PMID: 31462073

[ref30] Templin J. L. Henson R. A. (2006). Measurement of psychological disorders using cognitive diagnosis models. Psychol. Methods 11, 287–305. doi: 10.1037/1082-989X.11.3.287, PMID: 16953706

[ref31] Tu D. (2019). flflexCDMs. Available at: http://www.psychometrics-studio.cn (Accessed June 3, 2023).

[ref32] von Davier M. (2005). A general diagnostic model applied to language testing data. ETS Res. Rep. Ser. 2005, i–35. doi: 10.1002/j.2333-8504.2005.tb01993.x, PMID: 17535481

[ref33] Wardat Y. Belbase S. Tairab H. Takriti R. A. Efstratopoulou M. Dodeen H. (2022). The influence of school factors on students’ mathematics achievements in trends in international mathematics and science study (TIMSS) in Abu Dhabi emirate schools. Educ. Sci. 12:424. doi: 10.3390/educsci12070424, PMID: 37235093 PMC10206125

[ref34] Wu X. Wu R. Chang H. H. Kong Q. Zhang Y. (2020). International comparative study on PISA mathematics achievement test based on cognitive diagnostic models. Front. Psychol. 11:2230. doi: 10.3389/fpsyg.2020.02230 33013581 PMC7509072

